# Associations Between Immune Senescence and Immune Reconstitution Among People Living With HIV Undergoing Antiretroviral Therapy

**DOI:** 10.1155/jimr/8683624

**Published:** 2026-06-02

**Authors:** Zhe Qian, Huolan Long, Houji Wu, Jingfang Xie, Suling Chen, Jian Wu, Shaohang Cai

**Affiliations:** ^1^ Second Department of Elderly Respiratory, Guangdong Provincial People’s Hospital, Guangdong Academy of Medical Sciences, Guangdong Provincial Geriatrics Institute, Southern Medical University, Guangzhou, China, fimmu.com; ^2^ Department of Infectious Diseases, Nanfang Hospital, Southern Medical University, Guangzhou, China, fimmu.com; ^3^ Department of Geriatrics, Guangdong Provincial People’s Hospital, Guangdong Academy of Medical Sciences, Guangdong Provincial Geriatrics Institute, Guangzhou, China, gdghospital.org.cn

**Keywords:** AIDS, HIV, immune reconstitution, immune senescence

## Abstract

**Background:**

Failure of immune reconstitution (IR), characterized by suboptimal CD4^+^ T‐cell recovery despite virologic suppression on antiretroviral therapy (ART), remains a critical challenge for people living with human immunodeficiency virus (PLWHIV). The contribution of immune senescence to this failure is incompletely understood.

**Objective:**

This study aimed to determine the role of pre‐ART immune senescence characterized by lymphocyte profile and lymphocyte cellular senescence markers in nonimmune reconstitution (NIR) after 48 weeks of ART initiation.

**Methods:**

We first performed a SenMayo gene set score from publicly available CD4^+^ T‐cell scRNA‐seq data. Subsequently, a prospective cohort of 510 ART‐initiating PLWHIV was assessed at baseline and week 48. IR was strictly defined as a CD4^+^ T‐cell count increase over 350 cells/uL. Logistic regression; immunophenotyping; cytokine profiling, including TNF‐α, IFN‐γ and CD‐107a; and cellular senescence marker, including p16, p21, p53, and senescence‐associated β‐galactosidase assessments identified NIR factors.

**Results:**

Bioinformatics analysis revealed multiple ART‐naïve CD4^+^ T‐cell subtypes exhibited elevated cellular senescence scores. Patients with IR were significantly younger than those with NIR (both in AIDS and non‐AIDS subcohorts, *p*  < 0.05). Multivariable analysis indicated age was an independent risk factor for NIR. Before ART initiation, the NIR group exhibited a distinctly senescent immune profile characterized by reduced naïve T cells, increased terminally differentiated T cells, and heightened chronic inflammation with elevated cytokine secretion. ART failed to fully reverse these immune abnormalities, and cellular senescence was most pronounced in CD4^+^ T cells within the NIR group prior to treatment.

**Conclusion:**

Pre‐ART immune senescence is strongly associated with NIR in PLWHIV receiving ART. These findings necessitate considering immunological age and immune senescent status for personalized therapeutic strategies.

## 1. Introduction

HIV infection represents a significant global public health issue, leading to numerous fatalities worldwide [1]. The implementation of highly active antiretroviral therapy (HAART) in clinical settings has substantially improved the life expectancy of people living with HIV (PLWHIV) [2].

Upon HIV infection, the virus targets and gains entry into CD4^+^ T cells, initiating a cascade that promotes viral replication. This replication not only damages CD4^+^ T cells directly but also induces an immune response, exacerbating their loss. The resultant decline in CD4^+^ T cells compromises immunological defenses, elevating the risk of opportunistic infections and malignancies [3]. The advent of antiretroviral therapy (ART) has significantly suppressed HIV replication, enabling many PLWHIV to achieve immune reconstitution (IR) [4]. Nonetheless, a subset of PLWHIV remains unable to attain IR, even with ART support, with up to 30% failing to do so despite viral suppression [5, 6]. These data varied according to the different definitions of immune reconstitution used in each study.

Various factors contribute to the failure of IR in PLWHIV, including male sex and low nadir CD4^+^ T‐cell counts before ART [7]. Studies also indicated that body mass index (BMI) [8] and co‐infection with hepatitis C virus (HCV) [9] can influence IR outcomes post‐ART. Intriguingly, certain research findings suggested that T‐cell function, notably cofilin‐induced T‐cell motility for tissue repopulation, plays a crucial role in the regulation of immune reconstitution among PLWHIV [10, 11]. Interestingly, an increasing body of research indicated that immune aging/immune senescence may play a significant role in inadequate immune reconstitution. Moreover, immune senescence is linked to adverse clinical outcomes in PLWHIV, such as end‐stage organ failure and increased mortality [12–15]. However, the precise relationship between immune senescence and the lack of IR remains elusive.

The alterations within the immune system are frequently intricately interconnected. Immune senescence is the gradual deterioration of the immune system associated with natural aging [16]. In PLWHIV, this manifests as a distinct phenotype characterized by a decreased CD4/CD8 ratio, lower naïve/memory cell ratio, and increased CD8^+^ CD28^−^ cells [13]. The aging of the immune system, a debated phenomenon, is rooted in the concept of replicative senescence from cell biology. This describes how cells reach their division limit (Hayflick limit) and either undergo apoptosis or lose function. A key hallmark of immune senescence is a redistribution of T‐cell subpopulations, notably a reduction in naïve T cells and a compensatory proliferation of naïve into memory T cells as thymic activity wanes [17]. Indeed, many of the alterations that affect the immune system in PLWHIV are reminiscent of the process of immune aging, characteristic of old age. Upon the occurrence of HIV infection and the establishment of a persistent viral reservoir, the immune system is repeatedly and continuously activated at low intensities. This systemic immune activation characterized by persistent chronic inflammation results in extensive cellular turnover, cellular senescence, and apoptosis. Consequently, over time, this leads to the development of immune senescence and a progressive decline in regenerative capacity [18]. Such shifts are implicated in diverse clinical outcomes [19] and mirror aging phenotypes in the elderly [20]. These alterations also predispose PLWHIV to comorbidities often associated with immune senescence and inflammation [14].

Cellular senescence is an age‐related phenotype elicited by acute or chronic damage, and this phenomenon can be manifested in lymphocytes, too [21–23]. Unlike variations in the immune cell spectrum, the terminology of cellular senescence emphasizes a stable cellular proliferative arrest [21]. Cellular senescence was found in peripheral blood leukocytes and CD8^+^ T cells from PLWHIV [24, 25]. Former studies confirmed that HIV proteins Nef and Tat can induce mesenchymal stem cell (MSC) senescence and that there is an association between senescence, aging, and HIV comorbidities [26]. However, there are a lack of studies linking the phenomenon of cellular senescence in lymphocytes to immune recovery in PLWHIV.

To date, no studies have explicitly examined the association between immune senescence and immune reconstitution in PLWHIV. Through preliminary bioinformatics analysis, researchers found that HIV infection may cause CD4^+^ T cells to exhibit a significant cellular senescence phenotype. Thus, it was reasonable to hypothesize that the immune status of PLWHIV before ART is predictive of their immune reconstitution post‐ART. Additionally, we proposed that the immune status in PLWHIV may undergo dynamic changes after ART initiation. In light of these considerations, we designed a cohort study to explore the relationship between the baseline aging phenotype and the outcome of immune reconstitution in PLWHIV after 48 weeks of ART. We anticipate that PLWHIV with a senescent immune phenotype before ART exhibit less robust immune reconstitution than those with a healthier baseline immune status.

## 2. Methods

### 2.1. Study Design and Study Participants

This study was designed to (1) evaluate the impact of age on immune reconstitution, (2) examine the spectrum and functional characteristics of lymphocytes before and after initiation of HAART, and (3) investigate the role of cellular senescence, particularly in CD4^+^ T cells, in immune reconstitution. All participants were consecutively enrolled and regularly monitored at the Department of Infectious Diseases, NanFang Hospital, from January 2022 to December 2024. Inclusion criteria for participants were as follows: (1) confirmed diagnosis of HIV infection, (2) initiation of HAART and regular follow‐up, (3) no prior treatment with steroids or cytotoxic agents, and (4) absence of a history of organ transplantation, neoplastic diseases, or autoimmune disorders. Comprehensive medical records, including complete blood count (CBC), biochemical profiles, and HIV virological parameters, were systematically collected during each routine follow‐up visit. Participant retention throughout the follow‐up period may be enhanced through the optimization of the study design, the provision of logistical and financial support, and the application of flexible data collection methodologies. Finally, a total of 510 PLWHIV were enrolled for further evaluation.

This study was conducted in accordance with the ethical principles outlined by the responsible committee on human experimentation, *the Declaration of Helsinki*, and the guidelines of *Good Clinical Practice* (GCP). The study protocol was approved by the Ethics Committee of Nanfang Hospital (Approval Number: NFEC‐2021‐178). Prior to enrollment, all participants were thoroughly informed about the study’s objectives, potential risks, benefits, and the nature of their involvement. Written informed consent was obtained from all participants in accordance with ethical standards.

### 2.2. Participants and Demographics

A total of 510 PLWHIV were included in the final analysis. Participants were initially stratified by clinical stage at diagnosis: AIDS stage (*n* = 210) and non‐AIDS stage (*n* = 300). Subsequently, all participants were classified into an immunological responders (IR) group or a nonimmunological responders (NIR) group based on the achievement of IR after 48 weeks of ART. The resulting cohort distribution was as follows: AIDS stage (IR, *n* = 164; NIR, *n* = 46) and non‐AIDS stage (IR, *n* = 199; and NIR, *n* = 101). A statistically significant difference in mean age was observed between the groups, with the NIR group being older than the IR group in both the non‐AIDS (*p* = 0.029) and AIDS (*p* = 0.012) cohorts (Table 1).

**Table 1:** Baseline characteristics of PLWHIV in AIDS and Non‐AIDS stages.

Variables	PLWHIV in AIDS stages		*p*‐Value	PLWHIV in non‐AIDS stages		*p*‐Value
	NIR	IR		NIR	IR	
Sample size	46	164	—	101	199	—
Gender (male, %)	40 (87%)	146 (89%)	0.697	90 (89.1%)	189 (95%)	0.060
Age (years)	44.24 ± 11.48	39.11 ± 12.29	0.012	38.34 ± 13.00	35.06 ± 10.42	0.029
Height (cm)	165.69 ± 8.46	167.12 ± 7.25	0.259	167.54 ± 6.88	167.91 ± 7.48	0.675
Weight (kg)	58.67 ± 9.27	59.44 ± 10.98	0.669	60.52 ± 9.14	63.23 ± 13.75	0.044
Baseline CD4 (cells/μL)	115.83 ± 53.74	110.92 ± 57.78	0.606	297.80 ± 52.17	287.92 ± 57.51	0.148
Nadir CD4 (cells/μL)	62.24 ± 34.54	61.59 ± 39.28	0.507	89.68 ± 40.23	92.12 ± 44.55	0.463
Baseline CD8 (cells/μL)	1081.29 ± 2091.07	830.02 ± 670.67	0.431	1090.53 ± 550.41	1113.59 ± 587.72	0.743
Baseline CD4/CD8 ratio	0.20 ± 0.17	0.17 ± 0.13	0.301	0.34 ± 0.17	0.37 ± 0.80	0.727
HIV RNA viral load (Log_10_IU/mL)	4.47 ± 0.78	4.45 ± 0.73	0.864	4.10 ± 0.72	4.23 ± 0.64	0.126
WBC (×10^9^/L)	4.98 ± 1.72	5.04 ± 1.67	0.832	5.55 ± 1.55	5.77 ± 1.49	0.244
HGB (g/L)	130.67 ± 22.98	130.36 ± 24.82	0.938	143.86 ± 13.85	145.63 ± 14.46	0.311
PLT (×10^9^/L)	208.61 ± 86.66	196.64 ± 72.28	0.345	221.38 ± 59.87	219.87 ± 58.34	0.834
ALT (U/L)	25.10 ± 21.01	28.98 ± 29.63	0.407	25.48 ± 31.19	29.54 ± 46.92	0.435
CR (μmol/L)	76.91 ± 42.97	72.88 ± 16.74	0.335	75.44 ± 17.35	74.95 ± 12.28	0.777

Prior to participant recruitment, a power analysis was conducted using (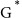
Power Version 3.1.9.7) to determine the minimum sample size required. Based on an estimated effect size, calculated from a previous study performed in the center, we set the significance level (α) at 0.05 and the statistical power (1‐β) at 0.80. The analysis indicated that a total of 16 subjects per group were required. To account for a projected dropout rate of 30%, we enrolled a final sample of 46 participants. We utilized stratified random sampling to select 23 patients from each group (AIDS stage and non‐AIDS stage) from the total population. These cohorts were matched for age and baseline cell counts. Detailed baseline characteristics for all participants are provided in Table S3.

### 2.3. AIDS Stage and Immune Reconstitution

According to the Chinese guidelines for the diagnosis and treatment of human immunodeficiency virus infection/acquired immunodeficiency syndrome [27], individuals receiving ART with peripheral blood viral load below the detection limit for 48 weeks and CD4^+^ T lymphocyte count still below 350 cells/uL for 24 and 48 weeks separately were defined as immune nonimmune responders (NIRs) [28]. The AIDS stage was defined as a positive HIV infection accompanied by a CD4^+^ T‐cell count of less than 200 cells/µl or the presence of related opportunistic infections at diagnosis [5]. The undetectable HIV viral load was defined as a level below the lower detection limit, specifically under 40 IU/mL.

### 2.4. Single‐Cell RNA Sequencing (scRNA‐seq) Data Analysis

Publicly available scRNA‐seq data derived from CD4^+^ T cells of people living with HIV (PLWHIV) were analyzed in this study [29]. The raw FASTQ data underwent initial processing, including quality control using FastQC, followed by read alignment and unique molecular identifier (UMI) quantification with CellRanger (Version 9.0.1). Downstream analyses were conducted in R using the Seurat package (Version 5.3.0). Standard quality control criteria were applied, including filtering of low‐quality cells, followed by data normalization and scaling. To mitigate potential batch effects across datasets, the Harmony integration algorithm was employed. Dimensionality reduction was performed using Principal Component Analysis (PCA), and the integrated data were visualized in two dimensions using Uniform Manifold Approximation and Projection (UMAP) and *t*‐distributed Stochastic Neighbor Embedding (*t*‐SNE). Clustering of cells was carried out on the integrated data, and cluster‐specific marker genes were identified using the FindAllMarkers function. Cell type classification and annotation were performed by comparing the expression profiles of the identified markers with canonical cell type markers referenced in the CellMarker database (http://xteam.xbio.top/CellMarker/). Finally, the SenMayo score, a predefined gene signature for cellular senescence, was used to quantify the level of cellular senescence within each cell cluster using the AddModuleScore function [30].

### 2.5. Laboratory Assays

All laboratory assessments were conducted in compliance with standard operating procedures (SOPs) and routine quality control measures at the Clinical Laboratory Department of Nanfang Hospital. Assays were performed strictly according to the manufacturers’ instructions. HIV‐specific antibodies were detected in patient serum samples via serological testing, adhering to the national HIV testing guidelines established by the National AIDS Control Organization (NACO). HIV‐1 viral load was quantified using the Abbott m2000 Real‐Time PCR system, with a lower limit of detection (LLOD) of 40 IU/mL. CD4^+^/CD8^+^ T‐cell counts were precisely measured using the BD FACSCount flow cytometry system. CBCs were analyzed using a Sysmex SE9000 automated hematology analyzer. Serum biochemical parameters, including alanine aminotransferase (ALT), aspartate aminotransferase (AST), and albumin (ALB), were assessed using the Olympus AU5400 automated chemistry analyzer within 2 h of sample collection.

### 2.6. Peripheral Blood Mononuclear Cell (PBMC) Isolation and Cryopreservation

PBMCs were isolated from whole blood via Ficoll–Hypaque density gradient centrifugation. Following isolation, the cell viability was assessed. PBMCs were then cryopreserved in liquid nitrogen using a freezing medium consisting of 90% fetal bovine serum (FBS) and 10% dimethyl sulfoxide (DMSO). The cells were subjected to controlled‐rate freezing by initially transferring them to a specialized container and cooling them to −80°C before transfer to a liquid nitrogen tank for long‐term storage.

### 2.7. Flow Cytometry Assays and Data Analysis

#### 2.7.1. Cell Staining and Acquisition

PBMCs, isolated from participants, were utilized for flow cytometric analysis. Cells were washed with phosphate‐buffered saline (PBS) containing 5% bovine serum albumin (BSA) and stained with LIVE/DEAD\texttrademark Fixable Viability Stain 506 (BD Biosciences, San Jose, CA, USA) to exclude nonviable cells. Subsequently, cells were labeled with specific fluorochrome‐conjugated surface antibodies. Detailed information regarding the antibody panels is provided in Table S1. Data acquisition was performed on a CytoFLEX flow cytometer (BF41495, Beckman).

#### 2.7.2. Flow Cytometric Data Analysis and Gating Strategy

Flow cytometry data were analyzed using FlowJo software (Version 10.8.1, BD Bioscience). Sequentially expressed cell surface markers were gated using the Fluorescence Minus One (FMO) control strategy to accurately define positive populations. The detailed gating strategies are presented in Figure S1.

#### 2.7.3. T‐Cell and B‐Cell Subpopulation Definitions

T‐cell subsets were identified and quantified based on the expression of surface markers: effector memory T cells (TEM, CCR7^−^CD45RA^−^), central memory T cells (TCM, CCR7^+^CD45RA^−^), and effector memory re‐expressing CD45RA T cells (TEMRA, CCR7^−^CD45RA^+^). The cytotoxic marker CD107a was also assessed. B‐cell subsets were defined using canonical phenotypic markers: memory B cells (CD27^+^CD38dim), memory regulatory B cells (CD24^+^CD27^+^), and regulatory B cells (CD24^+^CD38hi).

#### 2.7.4. Ex Vivo Stimulation and Intracellular Cytokine Staining (ICS)

PBMCs were stimulated ex vivo to assess both nonspecific and antigen‐specific cytokine secretion. Nonspecific stimulation: PBMCs were stimulated with phorbol 12‐myristate 13‐acetate (PMA, 100 ng/mL) (Invitrogen) and ionomycin (1 μg/mL) (Multisciences, China) for a total of 5 h. Brefeldin A was added after the first hour to inhibit the nonspecific secretion of TNF‐α, IFN‐γ. Antigen‐Specific Stimulation: PBMCs were stimulated with peptide pools (2 μg/mL per peptide) in the presence of costimulatory antibodies, anti‐CD28 and anti‐CD49d (1 μg/mL each; Invitrogen), for 1 h at 37°C in 5% CO_2_. Cells were then incubated for an additional 5 h with Brefeldin A (1:1000 dilution; Biolegend). Negative control: a control containing only anti‐CD28 and anti‐CD49d in the medium (with matched final DMSO concentration) was included to determine background activation. Positive control: PMA (100 ng/mL) and ionomycin (1 μg/mL) were used to confirm cellular responsiveness. Following stimulation, cells were washed, labeled with specific surface antibodies, and then fixed (Biolegend), permeabilized, and stained with fluorochrome‐conjugated monoclonal antibodies targeting intracellular TNF‐α and IFN‐γ. Frequencies of cytokine‐positive T cells in the peptide‐stimulated wells were background‐subtracted using the values from the matched negative controls. A response was considered positive if the background‐subtracted frequency exceeded 0.05% of CD4^+^ T or CD8^+^ T cells and was at least twofold above the background (irrelevant peptide stimulation).

### 2.8. Senescence‐Associated β‐Galactosidase (SA‐β‐gal) Assay

There were 22 and 23 samples of immune responders (IRs) and NIRs, respectively, performed that underwent senescence‐associated β‐galactosidase (SA‐β‐gal) staining. SA‐β‐gal activity in CD4^+^ T cells, CD8^+^ T cells, and CD19^+^ B cells was assessed using the SA‐β‐gal assay kit (Beyotime, C0602), following the manufacturer’s protocol. After three washes with PBS, cells were incubated with the SA‐β‐gal staining solution at 37°C overnight. The stained cells were then examined and quantified under a light microscope.

### 2.9. RNA Extraction and Quantitative Real‐Time Polymerase Chain Reaction (qRT‐PCR)

Total RNA was extracted from CD4^+^ T cells, CD8^+^ T cells, and CD19^+^ B cells using the TRIzol reagent (Invitrogen, Cat. Number 15596026) according to the manufacturer’s instructions. The purity and integrity of RNA were assessed using standard protocols. Total RNA was then reverse‐transcribed into complementary DNA (cDNA) utilizing the PrimeScript RT Master Mix (TaKaRa Biotechnology, Dalian, China). Quantitative real‐time PCR (qRT‐PCR) was performed on a LightCycler 480 II system (Roche Diagnostics, Basel, Switzerland) with the SYBR Green RT‐PCR Kit (TaKaRa Biotechnology). The mRNA expression levels of p21, p16, and p53 were detected. β‐actin (ACTB) mRNA expression was used as an internal control for the normalization of relative gene expression, with data analysis conducted using the 2^−ΔΔCt^ method. The specific primers, synthesized by Shenggong (Shanghai, China), are listed in Table S2. A total of 21 samples from the IR group and 19 samples from the NIR group were tested.

### 2.10. Western Blotting (WB)

CD4^+^ T cells, CD8^+^ T cells, and CD19^+^ B cells were lysed using radioimmunoprecipitation assay (RIPA) buffer (Beyotime, Cat. Number P0013B), supplemented with protease inhibitor phenylmethanesulfonylfluoride (PMSF; Beyotime, Cat. Number ST506) and phosphatase inhibitors (Beyotime, Cat. Number P1081) to extract total cellular proteins. Protein lysates were subjected to SDS‐PAGE electrophoresis, followed by electroblotting onto polyvinylidene fluoride (PVDF) membranes. Nonspecific binding was prevented by blocking the membranes with 5% BSA in Tris‐buffered saline containing 0.1% Tween 20 (TBST) at 37°C for 1 h. The membranes were incubated with primary antibodies overnight at 4°C. The protein expression levels of p21, p16, and p53 were detected. The following primary antibodies were used for WB: anti‐p16 (1:500, ab189034, Abcam), anti‐p21 (1:2000, 10355‐1‐AP, Proteintech), anti‐p53 (1:5000, 10442‐1‐AP, Proteintech), and anti‐β‐actin (1:1000, 20536‐1‐AP, Proteintech). After primary antibody incubation, the membranes were washed with TBST and subsequently incubated with secondary antibodies (goat antimouse, sc‐2005, Santa Cruz Biotechnology; goat antirabbit, sc‐2004, Santa Cruz Biotechnology) at room temperature for 1 h. Protein bands were visualized using enhanced chemiluminescence detection (ECL, Advance, Cat. Number RPN2235 GE Healthcare Life Sciences). Western blot experiments were performed in triplicate, and band intensities were quantified using ImageJ software (NIH, Bethesda, MD, USA). Relative protein levels were normalized to β‐actin to account for loading variations. A total of 23 samples from the IR group and 22 samples from the NIR group were tested. Detailed information regarding the antibody panels is provided in Table S1.

### 2.11. Statistical Analysis

Statistical analyses were conducted using the Statistical Package for the Social Sciences (SPSS, Version 20.0, IBM, Chicago, IL, USA) and GraphPad Prism (Version 8.0.2, San Diego, CA, USA). Continuous variables were assessed for normal distribution using the Shapiro–Wilk test. Data confirming a normal distribution were presented as mean ± standard deviation (SD) and were compared using the Student’s *t*‐test (for two groups) or one‐way analysis of variance (ANOVA, for three or more groups). Data that did not meet the assumption of normality were presented as the median and interquartile range (IQR) and compared using the Mann–Whitney *U* test or the Kruskal–Wallis *H* test, as appropriate. Categorical variables were reported as frequencies and percentages and compared using the chi‐square (*χ* ^2^) test. Univariate and multivariate binary logistic regression analyses were employed to identify independent factors associated with the outcomes of interest (dichotomous variable). Simple linear regression analysis was used to investigate the correlation and relationship between two continuous variables. All statistical tests were two‐tailed, and a *p*‐value of <0.05 was considered statistically significant.

## 3. Results

### 3.1. Single‐Cell Sequencing Reveals the Cellular Senescence Phenotype of CD4^+^ T Cells After HIV Infection

**Figure 1:**
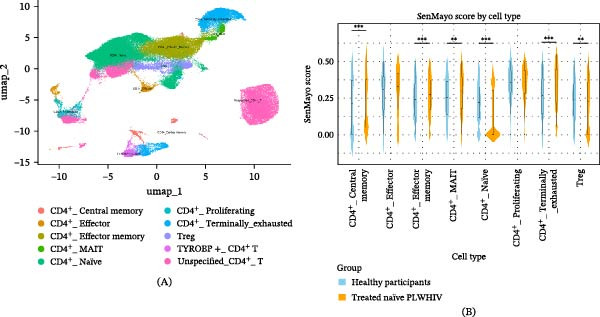
Characterization of CD4^+^ T‐cell profiles. (A) UMAP visualization of the CD4^+^ T‐cell subsets identified from the scRNA‐seq data of healthy participants (*n* = 4) and ART‐naïve PLWHIV (*n* = 14). The below panel details the specific cell subsets identified. (B) SenMayo scores on CD4^+^ T‐cell subtypes between the group of healthy participants and ART‐naïve PLWHIV.

scRNA‐seq was performed on a total of 13 CD4^+^ T cell samples, comprising four healthy control (HC) individuals and nine antiretroviral therapy (ART)‐naïve PLWHIVs. The baseline mean age was comparable between the HC group (28.75 years) and the ART‐naïve PLWHIV group (29.00 years). Integration and analysis of public scRNA‐seq databases identified 10 distinct clusters of CD4^+^ T cells (Figure 1A). Cellular senescence levels were quantified for eight of these classic clusters using the validated SenMayo geneset score [30], reported as median ± IQR. Notably, five CD4^+^ T cell subsets in the ART‐naïve PLWHIV cohort exhibited significantly higher SenMayo scores, representing elevated cellular senescence levels compared to HC. These clusters included CD4^+^ central memory cells (0.25 ± 0.32 vs. 0.21 ± 0.37), CD4^+^ effector memory cells (0.27 ± 0.21 vs. 0.24 ± 0.22), CD4^+^ mucosal‐associated invariant T (MAIT) cells (0.29 ± 0.22 vs. 0.25 ± 0.22), CD4^+^ naïve cells (0.22 ± 0.21 vs. 0.00 ± 0.30), and CD4^+^ terminally exhausted cells (0.34 ± 0.23 vs. 0.26 ± 0.24). Conversely, CD4^+^ regulatory T (Treg) cells in the ART‐naïve PLWHIV group demonstrated lower SenMayo scores (0.24 ± 0.22) compared to those of HC (0.23 ± 0.36) (Figure 1B). The specific SenMayo genes that are most differentially expressed between HCs and PLHIV within key cell subsets are shown in Figure S2. Consequently, we hypothesize that the heterogeneity of pre‐ART immune senescence profiles serves as a critical determinant of post‐ART immune reconstitution kinetics and magnitude.

### 3.2. Factors Related With Immune Reconstitution Among PLWHIV in AIDS Stage and Non‐AIDS Stage

**Figure 2:**
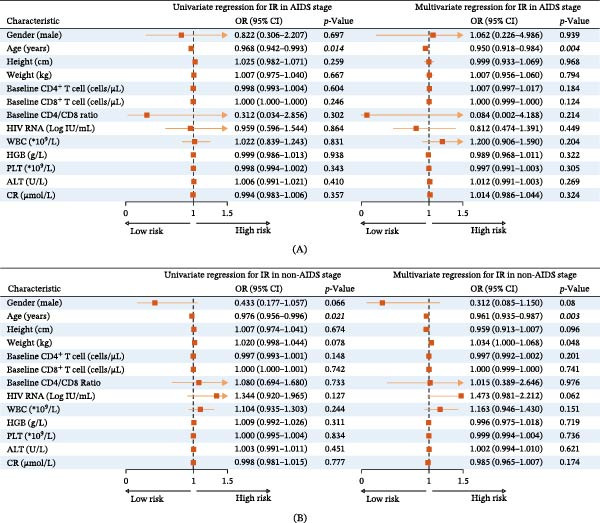
Univariate and multivariate analysis for immune reconstitution among PLWHIV. (A) Univariate analysis and multivariate analysis for immune reconstitution among PLWHIV in AIDS stage (*n* = 210, NIR: 46; IR: 164). (B) Univariate analysis and multivariate analysis for immune reconstitution among PLWHIV in the non‐AIDS stage (*n* = 300, NIR: 101; IR: 199). Abbreviations: ALT, Alanine transaminase; CR, Creatinine; HGB, Hemoglobin; HIV, Human immunodeficiency virus; IR, Immune reconstitution; PLT, Platelet; PLWHIV, People living with HIV; WBC, White blood cells.

Age was identified as a significant independent influencing factor for IR at week 48 among PLWHIV in the AIDS stage, evidenced in both univariate analysis (OR: 0.968; 95% CI: 0.942–0.993; *p*  = 0.014) and multivariate analysis (OR: 0.950; 95% CI: 0.918–0.984; *p*  = 0.004) (Figure 2A). Similarly, in the non‐AIDS stage cohort, age also independently influenced IR, as demonstrated in the univariate analysis (OR: 0.976; 95% CI: 0.956–0.996; *p*  = 0.021) and multivariate analysis (OR: 0.961; 95% CI: 0.935–0.987; *p*  = 0.003) (Figure 2B).

### 3.3. Dynamic Changes of CD4^+^ T Cell and HIV Viral Load in PLWHIV

**Figure 3:**
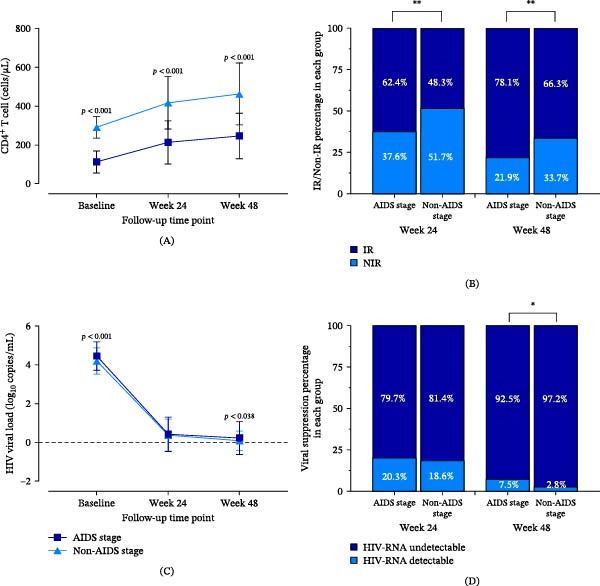
The changes in CD4^+^ T‐cell counts and HIV viral load from ART initiation to week 48. (A) Dynamic changes for CD4^+^ T cells following HAART in PLWHIV of AIDS stage and non‐AIDS stages (baseline: 291.24 ± 55.87 vs. 111.99 ± 56.83, *n* = 510, AIDS stage: 210, non‐AIDS stage: 300, *p* < 0.001; week 24:416.50 ± 135.56 vs. 212.43 ± 110.44, *n* = 493, AIDS stage: 203, non‐AIDS stage: 290, *p* < 0.001; week 48:461.86 ± 159.69 vs. 245.63 ± 117.53, *n* = 491, AIDS stage: 201, non‐AIDS stage: 290, *p* < 0.001). (B) The proportion of PLWHIV of non‐AIDS stage and AIDS stage achieved IR at week 24 and week 48. (C) Dynamic changes for HIV viral load following HAART in PLWHIV of AIDS stage and non‐AIDS stages (baseline: 4.19 ± 0.68 vs. 4.45 ± 0.73, *n* = 480, AIDS stage: 199, non‐AIDS stage: 281, *p* < 0.001; week 24:0.37 ± 0.82 vs. 0.41 ± 0.89, *n* = 475, AIDS stage: 197, non‐AIDS stage: 278, *p* < 0.001; week 48 : 0.08 ± 0.50 vs. 0.22 ± 0.85, *n* = 482, AIDS stage: 199, non‐AIDS stage: 283, *p* < 0.001). (D) The proportion of PLWHIV of non‐AIDS stage and AIDS stage achieved viral suppression at week 24 and week 48.

We comprehensively analyzed the dynamic changes in CD4^+^ T‐cell counts and HIV viral load from the initiation of ART to week 48 (Figure 3). Significant recovery of CD4^+^ T‐cell counts was observed in both the non‐AIDS and AIDS stage groups post‐ART, and CD4^+^ T‐cell counts in the non‐AIDS group were consistently higher than those in the AIDS group at both week 24 (*p* < 0.001) and week 48 (*p* < 0.001) (Figure 3A). Additionally, both groups substantially reduced viral load following ART initiation. Notably, the non‐AIDS stage group exhibited a lower viral load compared to the AIDS stage group at week 48 (*p* = 0.038) (Figure 3C).

Furthermore, we observed a higher proportion of PLWHIV in the AIDS stage group achieving IR at week 24 (62.4% vs. 48.3%, *p* < 0.01) and week 48 (78.1% vs. 66.3%, *p* < 0.01) (Figure 3B). Notably, a greater percentage of individuals in the non‐AIDS stage group achieved an undetectable HIV RNA viral load compared to the AIDS group at week 48 (97.2% vs. 92.5%, *p* < 0.05) (Figure 3D).

### 3.4. Age Was Related to Immune Senescence Among PLWHIV

**Figure 4:**
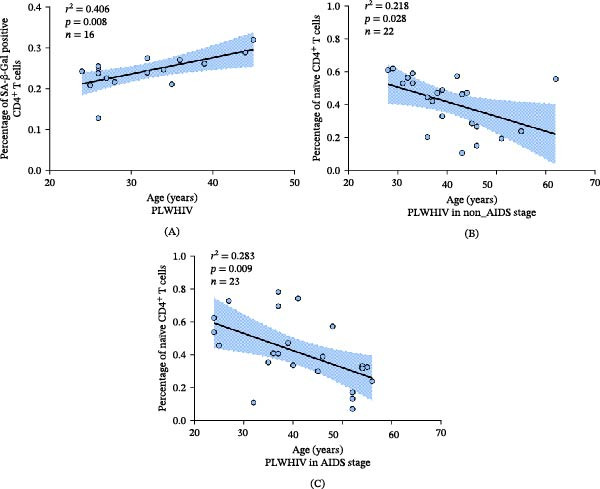
The relationship between age and immune senescent phenotype of lymphocytes. (A) The relationship between age (years) and percentage of SA‐β‐Gal positive CD4^+^ T cells among PLWHIV (*n* = 16; *r* ^2^ = 0.406; *p* = 0.008). (B) The relationship between age (years) and percentage of naïve CD4^+^ T cells among PLWHIV in the non‐AIDS stage (*n* = 22; *r* ^2^ = 0.218; *p* = 0.028). (C) The relationship between age (years) and percentage of naive CD4^+^ T cells among PLWHIV in AIDS stage (*n* = 23; *r* ^2^ = 0.283; *p* = 0.009). Abbreviations: AIDS, Acquired immunodeficiency syndrome; PLWHIV, People living with HIV.

Further, we explored the relationship between age and CD4^+^ T‐cell senescence phenotype (Figure 4). We observed a positive linear relationship between age and the percentage of SA‐β‐Gal positive CD4^+^ T cells among PLWHIV (*r* ^2^ = 0.406, *p* = 0.008) (Figure 4A). Additionally, a negative relationship was identified between age and the percentage of naïve CD4^+^ T cells in both the non‐AIDS (*r* ^2^ = 0.218, *p* = 0.028) (Figure 4B) and AIDS groups (*r* ^2^ = 0.283, *p* = 0.009) (Figure 4C). Given the potential association between age and the senescent phenotype of CD4^+^ T cells, we hypothesized that there may be differences in this immune senescence phenotype between the IR and NIR groups of PLWHIV receiving ART.

### 3.5. NIR Groups Had an Aging Immune Profile Before HAART

**Figure 5:**
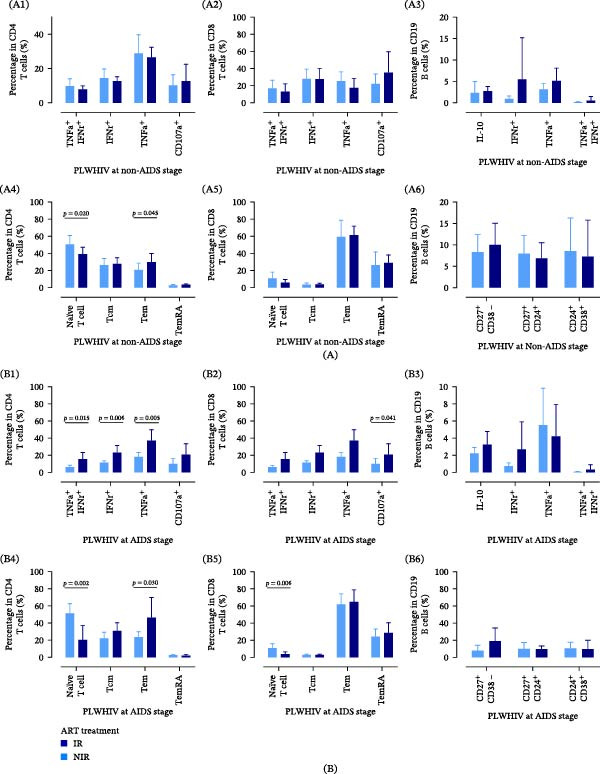
The baseline lymphocyte profile of IR and NIR PLWHIV in the non‐AIDS stage and AIDS stage, respectively. (A1) The percentage in CD4 T cells (%) with inflammation secretion phenotype among PLWHIV at non‐AIDS stage. (A2) The percentage in CD8 T cells (%) with inflammation secretion phenotype among PLWHIV at non‐AIDS stage. (A3) The percentage in CD19 B cells (%) with inflammation secretion phenotype among PLWHIV at non‐AIDS stage. (A4) The percentage in subtypes of CD4 T cell (%) among PLWHIV at non‐AIDS stage. (A5) The percentage in subtypes of CD8 T cell (%) among PLWHIV at non‐AIDS stage. (A6) The percentage in subtypes of CD19 B cell (%) among PLWHIV at non‐AIDS stage. (B1) The percentage in CD4 T cells (%) with inflammation secretion phenotype among PLWHIV at AIDS stage. (B2) The percentage in CD8 T cells (%) with inflammation secretion phenotype among PLWHIV at AIDS stage. (B3) The percentage in CD19 B cells (%) with inflammation secretion phenotype among PLWHIV at AIDS stage. (B4) The percentage in subtypes of CD4 T cell (%) among PLWHIV at AIDS stage. (B5) The percentage in subtypes of CD8 T cell (%) among PLWHIV at AIDS stage. (B6) The percentage in subtypes of CD19 B cell (%) among PLWHIV at AIDS stage. AIDS, acquired immunodeficiency syndrome; ART, antiretroviral therapy; HIV, human immunodeficiency virus; IR, immune reconstitution; NIR, nonimmune reconstitution; PLWHIV, people living with HIV.

During ART, we comprehensively assessed the lymphocyte subtypes in PLWHIV (Figure 5). Among non‐AIDS stage participants, distinct differences in CD4^+^ T‐cell subpopulations were observed between the IR and NIR groups. The NIR group exhibited a lower proportion of CD4^+^ naïve T cells (50.42 ± 10.38 vs. 38.96 ± 7.92, *n* = 16, *p* = 0.020) and a higher proportion of CD4^+^ Tem cells (20.78 ± 7.89 vs. 28.89 ± 9.94, *n* = 17, *p* = 0.045) compared to the IR group (Figure 5A4). However, significant disparities in cytokine secretion and cytotoxic functions among CD4^+^ T cells, CD8^+^ T cells, and CD19^+^ B cells were not observed between the IR and NIR groups at the non‐AIDS stage (Figure 5A1–A3), and also no significant differences in subpopulations were observed in CD8^+^ T cells and CD19^+^ B cells (Figure 5A5, A6).

In individuals diagnosed at the AIDS stage initially, significant variations in CD4^+^ T‐cell subpopulations were noted. The NIR group had reduced proportions of CD4^+^ naïve T cells (51.21 ± 11.32 vs. 20.38 ± 16.79, *n* = 17, *p* = 0.002) (Figure 5B4) and CD8^+^ naïve T cells (10.88 ± 5.07 vs. 3.86 ± 2.69, *n* = 17, *p* = 0.006) (Figure 5B5) compared to the IR group, as well as a higher proportion of CD4^+^ Tem cells (23.70 ± 6.22 vs. 46.38 ± 23.40, *n* = 16, *p* = 0.030) (Figure 5B4). Notably, cytokine secretion and cytotoxic functions of lymphocytes in the IR group were diminished relative to those in the NIR group during the AIDS stage. Specifically, percentages of IFNγ + TNFα+ CD4^+^ T cells (6.28 ± 2.02 vs. 15.50 ± 7.67, *n* = 18, *p* = 0.015), IFNγ+ CD4^+^ T cells (11.63 ± 6.98 vs. 23.05 ± 8.44, *n* = 16, *p* = 0.006), and TNFα+ CD4^+^ T cells (18.33 ± 4.85 vs. 37.28 ± 12.55, *n* = 18, *p* = 0.005) were significantly higher in the NIR group (Figure 5B1). Furthermore, the percentage of CD107a+ CD8^+^ T cells was also greater in the NIR group (9.84 ± 6.34 vs. 20.86 ± 12.62, *n* = 17, *p* = 0.041) (Figure 5B2). The cytokine secretion and cytotoxic functions and subpopulations were not significantly different in CD19^+^ B cells between the IR and NIR groups (Figure 5B3, B6).

### 3.6. HAART did Not Fully Restore Immune Function of Lymphocytes From IR and NIR Groups

**Figure 6:**
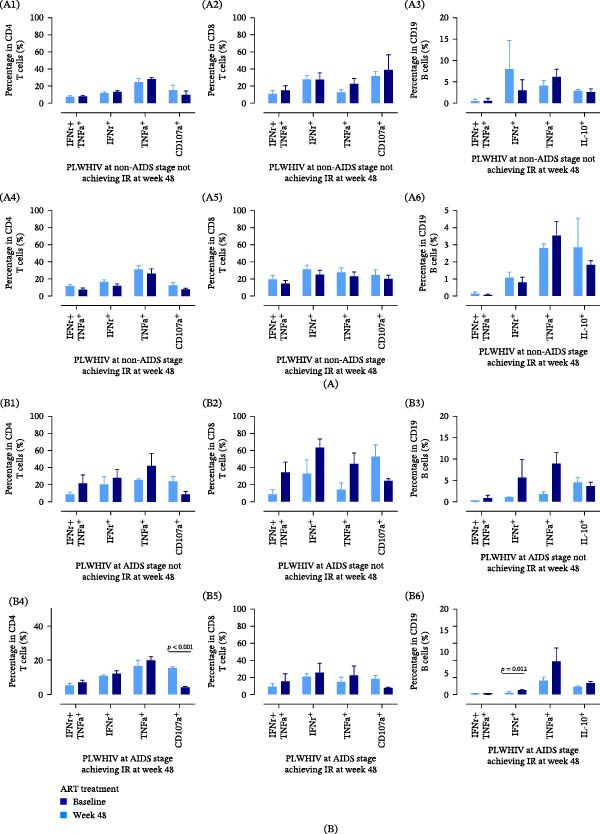
The dynamics of the lymphocyte profile of IR and NIR PLWHIV in the non‐AIDS stage and AIDS stage. (A1) The dynamics of percentage in CD4 T cells (%) of PLWHIV at non‐AIDS stage not achieving IR in week 48. (A2) The dynamics of percentage in CD8 T cells (%) of PLWHIV at non‐AIDS stage not achieving IR in week 48. (A3) The dynamics of percentage in CD19 B cells (%) of PLWHIV at non‐AIDS stage not achieving IR in week 48. (A4) The dynamics of percentage in CD4 T cells (%) of PLWHIV at non‐AIDS stage achieving IR in week 48. (A5) The dynamics of percentage in CD8 T cells (%) of PLWHIV at non‐AIDS stage achieving IR in week 48. (A6) The dynamics of percentage in CD19 B cells (%) of PLWHIV at non‐AIDS stage achieving IR in week 48. (B1) The dynamics of percentage in CD4 T cells (%) of PLWHIV at AIDS stage not achieving IR in week 48. (B2) The dynamics of percentage in CD8 T cells (%) of PLWHIV at AIDS stage not achieving IR in week 48. (B3) The dynamics of percentage in CD19 B cells (%) of PLWHIV at AIDS stage not achieving IR in week 48. (B4) The dynamics of percentage in CD4 T cells (%) of PLWHIV at AIDS stage achieving IR in week 48. (B5) The dynamics of percentage in CD8 T cells (%) of PLWHIV at AIDS stage achieving IR in week 48. (B6) The dynamics of percentage in CD19 B cells (%) of PLWHIV at AIDS stage achieving IR in week 48. AIDS, acquired immunodeficiency syndrome; ART, antiretroviral therapy; HIV, human immunodeficiency virus; IR, immune reconstitution; NIR, nonimmune reconstitution; PLWHIV, people living with HIV.

Subsequently, a comparative analysis was performed to assess cytokine secretion and cytotoxic functions of lymphocytes in PLWHIV at week 48, distinguishing between those in the non‐AIDS and AIDS stages (Figure 6). For the non‐AIDS stage group, no significant differences were observed in immune functions of lymphocytes between baseline and the week 48 assessment, irrespective of IR achievement (Figure 6A1–A6). For PLWHIV at the AIDS stage not achieving IR, ART was proved not to restore immune function of CD4^+^ T cells, CD8^+^ T cells, and CD19^+^ B cells (Figure 6B1–B3). The similar findings in the AIDS stage group achieving IR were confirmed except for a slight reduction in the percentage of CD107a+ CD4^+^ T cells (15.53 ± 1.50 vs. 4.14 ± 0.95, *n* = 17, *p* < 0.001) (Figure 6B4) after 48 weeks of ART and an increase in the percentage of IFNγ+ CD19^+^ B cells (0.43 ± 0.21 vs. 1.05 ± 0.13, *n* = 16, *p* = 0.012) (Figure 6B6).

### 3.7. Cellular Senescent Lymphocytes of NIR Groups

**Figure 7:**
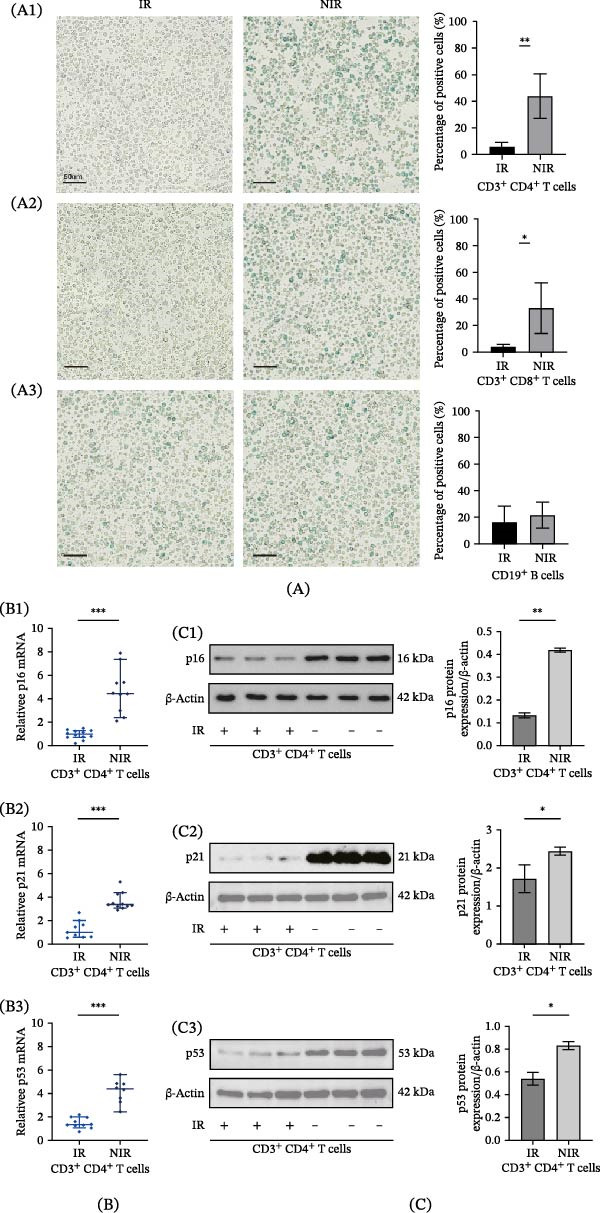
Cellular senescence in CD4^+^ T cells of the NIR group. Representative image of SA‐β‐gal staining in (A1) CD3^+^ CD4^+^ T cells (43.82 ± 16.71 vs. 5.21 ± 3.67, IR: 22, NIR: 23, *p* < 0.01), (A2) CD3^+^ CD8^+^ T cells (32.68 ± 12.92 vs. 3.21 ± 2.11, IR: 22, NIR: 23, *p* < 0.05), and (A3) CD19^+^ B cells from IR and NIR groups at baseline (21.37 ± 9.87 vs. 15.67 ± 12.32, IR: 22, NIR: 23, *p* = 0.19) (scale bars = 50 μm). (qRT‐PCR analysis of mRNA levels of (B1) p16 (4.67 ± 1.93 vs. 0.95 ± 0.39, IR:21, NIR: 19, *p* < 0.001), (B2) p21 (3.62 ± 0.72 vs. 1.25 ± 0.74, IR: 21, NIR: 19, *p* < 0.001), and (B3) p53 (4.19 ± 1.01 vs. 1.49 ± 0.46, IR: 21, NIR: 19, *p* < 0.001) in CD3^+^ CD4^+^ T cells from IR and NIR group. Western blotting (WB) analysis of protein levels of (C1) p16 (0.42 ± 0.01 vs. 0.13 ± 0.01, IR: 23, NIR: 22, *p* < 0.01), (C2) p21 (2.44 ± 0.10 vs. 1.72 ± 0.37, IR: 23, NIR: 22, *p* < 0.05), and (C3) p53 (0.83 ± 0.03 vs. 0.54 ± 0.06, IR: 23, NIR: 22, *p*  < 0.05) in CD3^+^ CD4^+^ T cells from IR and NIR groups.

After confirming the presence of an immune senescence phenotype in the NIR group, we further explored which lymphocyte subsets exhibited the most pronounced senescent features that might impact immune reconstitution. A higher proportion of SA‐β‐gal positive CD3^+^ CD4^+^ T cells was in the NIR group than in the IR group (43.82 ± 16.71 vs. 5.21 ± 3.67, *p* < 0.01) (Figure 7A1). The same findings were confirmed in CD3^+^ CD8^+^ T cells (32.68 ± 12.92 vs. 3.21 ± 2.11, *p* < 0.05) (Figure 7A2). However, the proportions of SA‐β‐gal positive CD19^+^ B cells were not significantly different between the NIR and IR groups (21.37 ± 9.87 vs. 15.67 ± 12.32, *p* = 0.19) (Figure 7A3).

Further, the mRNA levels of p16 (4.67 ± 1.93 vs. 0.95 ± 0.39, *p*  < 0.001), p21 (3.62 ± 0.72 vs. 1.25 ± 0.74, *p* < 0.001), and p53 (4.19 ± 1.01 vs. 1.49 ± 0.46, *p* < 0.001) were higher in CD3^+^ CD4^+^ T cells from the NIR group at baseline (Figure 7B1–B3). In addition, the protein expression levels of p16 (0.42 ± 0.01 vs. 0.13 ± 0.01, *p* < 0.01), p21 (2.44 ± 0.10 vs. 1.72 ± 0.37, *p* < 0.05), and p53 (0.83 ± 0.03 vs. 0.54 ± 0.06, *p* < 0.05) were also higher in NIR group than IR group (Figure 7C1–C3).

## 4. Discussion

Our findings from this study can be summarized on two levels. Demographically, an older age emerged as a factor affecting immune reconstitution. Then, through our bioinformatics analysis, we predicted that specific CD4^+^ T‐cell subsets, particularly effector memory and terminally differentiated cells, display a senescent transcriptional profile during HIV infection. This prediction was subsequently validated at the mRNA, protein, and cellular level in our prospective cohort, where we observed accumulation of these subsets (Figure 5) and elevated p16/p21 expression (Figure 7) in CD4^+^ T cells from the NIR group.

Despite the average age of PLWHIV not aligning with the definition of the elderly demographic, our study found that younger participants were more likely to achieve IR post‐ART, a finding corroborated by logistic regression analysis. However, in our study, the odds ratio for age concerning the failure of immune reconstitution was ~1, indicating that age may not be one of the predominant factors contributing to the overall failure of immune reconstitution. A study conducted in China showed that a total of five factors, including baseline CD4 T‐cell counts, age at ART initiation, BMI, herpes zoster infection, and total bilirubin, combined to influence the outcome of immune reconstitution in PLWHIV, and the odds ratio for failure of IR was 1.028 (95% CI was 1.020–1.037) [28]. In fact, the relationship between advancing chronological age and immune senescence is not linear [31]. Multiple factors contributed to the development of immune senescence during ART in PLWHIV, with natural aging being only one of these factors [32]. Therefore, it is essential to further investigate the role of immune senescence in the failure of immune reconstitution.

In the SenMayo senescence score analysis applied to CD4^+^ T‐cell subtypes, we observed that many CD4^+^ T‐cell subsets in ART‐naïve PLWHIV exhibited elevated cellular senescence scores, with the notable exception of regulatory T cells (Tregs). During HIV infection, Tregs play a complex and dual role in immune regulation. A hallmark of HIV infection is chronic systemic immune hyperactivation, which creates a persistent inflammatory milieu that accelerates CD4^+^ T‐cell depletion and contributes to tissue damage [33]. Tregs exert immunosuppressive effects on effector T cells (Teffs) and antigen‐presenting cells (APCs) through the secretion of inhibitory cytokines (e.g., IL‐10 and TGF‐β) as well as direct cell‐to‐cell contact mechanisms (e.g., involving CTLA‐4) [34]. Conversely, Tregs also suppress the proliferation and function of HIV‐specific CD8^+^ T cells (cytotoxic T lymphocytes). This inhibition impairs the host’s capacity to fully eliminate the virus, thereby promoting persistent viral replication and the establishment of chronic infection [35]. Furthermore, as Tregs themselves constitute a subset of CD4^+^ T cells, they are also susceptible to direct HIV infection and depletion [36]. A reduced SenMayo senescence score in regulatory T cells (Tregs) may indicate that these cells remain in a nonsenescent state, allowing them to exert a continuing suppressive influence during HIV infection and progression.

Age‐related changes in the human immune system, known as immune senescence, predominantly involve shifts in T‐cell subset distribution and function, including increased CD8 T cells and memory cell expansion, termed “memory inflation” [15]. Similar immune phenotypes to those found in older adults have been observed in PLWHIV [37]. Additionally, a previous study tracking naïve and memory CD4^+^ T‐cell trajectories in PLWHIV suggested that advanced age might significantly affect long‐term CD4^+^ T‐cell recovery [38]. Consequently, it is plausible that the confluence of aging and HIV infection contributes to the observed senescence variations.

As ART progressed, both the non‐AIDS and AIDS stage groups of PLWHIV exhibited general recovery in CD4^+^ T‐cell counts and a decrease in HIV viral load. Notably, individuals diagnosed in the non‐AIDS stage showed significantly higher rates of CD4^+^ T‐cell recovery and more effective viral suppression, aligning with previous findings [39]. Building on these insights, we further assessed lymphocyte subphenotypes and functions using flow cytometry for groups achieving or not achieving IR.

When evaluating CD4^+^ T‐cell subphenotypes, we observed that PLWHIV at the non‐AIDS stage achieving IR at week 48 had a lower proportion of CD4^+^ Tem cells and a higher proportion of CD4^+^ naïve T cells compared to those who did not achieve IR, aligning with previously reported immune senescence phenotypes in PLWHIV [40, 41]. Continuous HIV infection has been confirmed to induce a state of replicative senescence in vivo [42]. Notably, no significant differences were found in the proportions of CD8^+^ T‐cell and CD19^+^ B‐cell subtypes nor in the functional capacities of these cells between the AIDS and non‐AIDS group, regardless of whether it achieves IR or not. Nevertheless, at the AIDS stage, both CD4^+^ and CD8^+^ T cells in the NIR group exhibited typical senescent phenotypes, with notably higher proportions of cytokine‐secreting CD4^+^ T cells and cytotoxic CD8^+^ T cells. This could lead to the occurrence of chronic inflammation, a characteristic of HIV infection and a recognized mechanism for CD8^+^ T‐cell aging [43]. Nevertheless, the enhanced functionality of lymphocytes in the NIR group can be considered indicative of ongoing chronic immune activation and inflammation, both of which are established drivers of immune senescence and inflammaging [44]. This chronic activation likely propels T cells toward terminal differentiation (TemRA) and eventual proliferative exhaustion/senescence [45]. The interplay between chronic inflammation and immune senescence during HIV infection constitutes a noteworthy and rapidly evolving research domain within the field [46].

In fact, stem cell exhaustion is one of the hallmarks of aging, which was closely associated with tissue repair upon injury [47]. This situation can be easily understood in the context of HIV infection, which was a common stimulating factor for immune system, and the immune reconstitution was regarded as a tissue renewal/repair process upon HIV infection. Our study demonstrated that NIR PLWHIV continues to exhibit a senescent lymphocyte spectrum.

In addition to the senescent immune cell spectrum, lymphocytes themselves show a cellular senescence phenotype. The most salient feature of cellular senescence is a stable proliferative arrest mediated by the activation of the tumor suppressors TP53 and CDKN2A/p16 and their downstream effectors CDKN1A/p21. Together, these proteins inhibit cyclin‐dependent kinases (CDKs) and transcriptional activators (E2F family) that drive the cell cycle [21]. Former studies indicated that progression of HIV replication and immune evasion leads to the activation of cellular senescence pathways [32, 48].

However, it is not known whether cellular senescence, especially in lymphocytes, has an impact on immune reconstitution in PLWHIV. Our research confirmed that cellular senescence already exists in CD4^+^ T cells and CD8^+^ T cells from NIR PLWHIV before HAART, proved by higher levels of lysosomal senescence‐associated beta‐galactosidase (SA‐β‐Gal). Furthermore, we detected higher expression levels of p53‐, p16‐, and p21‐related mRNAs and proteins in CD4^+^ T cells, which were the targeted lymphocytes under HIV attack. As is all known, senescent cells were in a significant proliferative arrest state, so it may partially explain the immune reconstitution failure in PLWHIV. Luckily, this finding may provide a potential strategy to intervene in immune recovery with senolytics targeted at cellular senescent cells.

The factors affecting IR are multifaceted, including decreased bone marrow hematopoiesis, reduced thymic output leading to diminished de novo synthesis of naïve CD4^+^ T cells [49], ongoing virus replication, abnormal immune activation, alterations in cytokine secretion, and particular genetic or metabolic traits [50]. In our study, when we revisited the variation of the immune system that occurs after HIV infection by bringing them into the perspective of aging, we found that (1) stem cell depletion and (2) immune cell senescence, as typical hallmarks of aging, both play important roles in the process of immune reconstitution in PLWHIV. Immune senescence not only affects immune reconstitution in PLWHIV but also the development of important HIV comorbidities such as cardiovascular disease [51–53], chronic obstructive pulmonary diseases [54–57], and type 2 diabetes mellitus [58, 59]. Therefore, it is believed that intervention strategies targeting immune senescence in PLWHIV can not only restore their level of immune reconstitution but also significantly improve their quality of life.

In summary, our study reveals that the pretreatment immune senescence status significantly influences the immune response post‐ART initiation in PLWHIV. This immune senescence is multifactorial, emerging from a combination of advanced age and the HIV infection itself. Identifying the precise factors contributing to the varied levels of immune senescence in PLWHIV requires further detailed research for definitive conclusions. Despite these complexities, our findings highlight the critical importance of early ART initiation, especially for individuals in the non‐AIDS stage, emphasizing the necessity of timely intervention in HIV management.

However, there were several limitations in this study. First, the mechanistic component was constrained by a relatively small sample size, which may limit the generalizability of our findings. Second, as this study is primarily observational in nature, the results demonstrate correlational relationships rather than establishing direct causal associations.

## 5. Conclusion

Older age and pretreatment immune senescence status are significantly associated with immune reconstitution in PLWHIV following antiretroviral therapy initiation.

**Nomenclature**
AIDS: Acquired immunodeficiency syndrome
ART: Antiretroviral therapy
CBC: Complete blood counts
CI: Confidence interval
ART: Highly active antiretroviral therapy
HIV: Human immunodeficiency virus
IR: Immune reconstitution
NIR: Nonimmune reconstitution
PLWHIV: People living with HIV.

## Author Contributions

Conceptualization: Shaohang Cai and Jian Wu. Data curation, methodology: Huolan Long. Formal analysis: Zhe Qian and Suling Chen. Funding acquisition: Jingfang Xie and Shaohang Cai. Investigation: Houji Wu. Resources: Shaohang Cai. Supervision: Shaohang Cai, Jian Wu, and Jingfang Xie. Writing – original draft: Zhe Qian and Suling Chen.

## Funding

The studies were supported by the National Natural Science Foundation of China (Grant 82500570), the Guangdong Basic and Applied Basic Research Fund (Natural Science Foundation of the Guangdong Province) (Grant 2026A1515010711), and the Guangzhou Science and Technology Plan Project (2025 Basic and Applied Basic Research Project, Grant 2025A04J4146).

## Disclosure

All authors reviewed and approved the final manuscript. The authors reviewed and edited the content as needed and take full responsibility for the content of the publication. The funding sources did not have any influence on the study design; data collection; analysis and interpretation of the data; writing of the manuscript; or decision to submit for publication.

## Ethics Statement

This retrospective study was approved by the Ethics Committee of Nanfang Hospital (NFEC‐2021‐178), and the study protocol was performed in accordance with the Helsinki Declaration of 1964 and its later amendments. Informed consent was obtained from all individuals.

## Conflicts of Interest

The authors declare no conflicts of interest.

## Supporting Information

Additional supporting information can be found online in the Supporting Information section.

## Supporting information


**Supporting Information** Figure S1: Schematic diagram for flow cytometry gating strategy for T‐cell subset analysis. Figure S2 Genes from SenMayo Geneset expression levels on multiple CD4^+^ T‐cell subtypes split by HIV^+^ treated‐naïve and healthy individuals. Table S1: Antibody panels. Table S2: Primer sequences for quantitative real‐time PCR (from 5′ to 3′). Table S3: Baseline Characteristics of PLWHIV for mechanical experiment.

## Data Availability

The data supporting the findings of this study are available upon request from the corresponding author.
