# Supplementary material for: Associations Between Immune Senescence and Immune Reconstitution Among People Living With HIV Undergoing Antiretroviral Therapy
Source: J Immunol Res. 2026 Jun 2;2026:8683624. doi: 10.1155/jimr/8683624 (PMC13239133; doi:10.1155/jimr/8683624)
Supplement: Supplementary file 1 — Supporting Information Figure S1: Schematic diagram for flow cytometry gating strategy for T‐cell subset analysis. Figure S2 Genes from SenMayo Geneset expression levels on multiple CD4+ T‐cell subtypes split by HIV+ treated‐naïve and healthy individuals. Table S1: Antibody panels. Table S2: Primer sequences for quantitative real‐time PCR (from 5′ to 3′). Table S3: Baseline Characteristics of PLWHIV for mechanical experiment. [file JIMR-2026-8683624-s001.docx]

**Supplementary Figure 1** Schematic diagram for flow cytometry gating strategy for T cell subset analysis


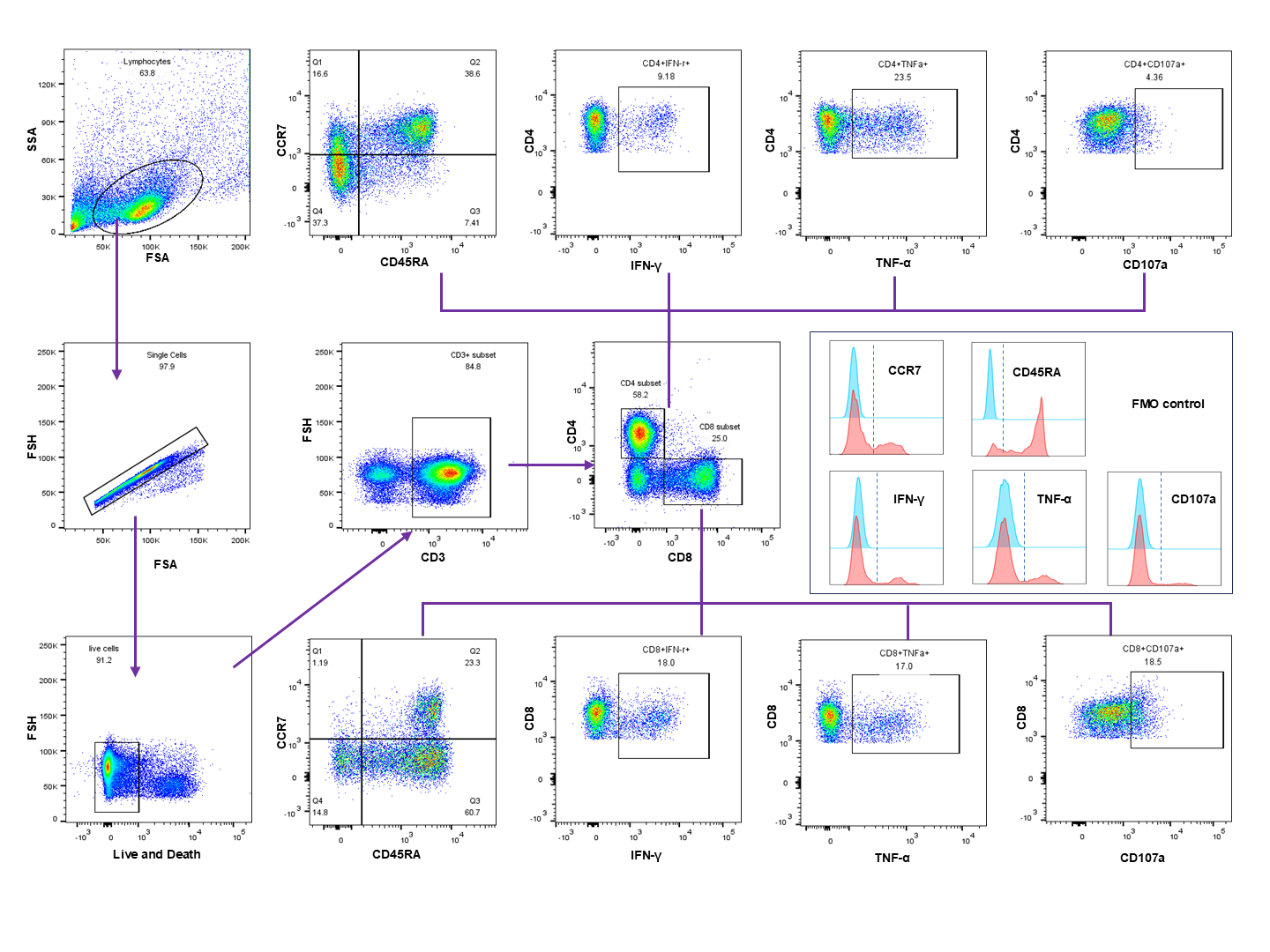


**Supplementary Table 1** Antibody panels

| **Antibody names** | **Fluorescent substance** | **Catalog** | **Manufacturer** | **Laser** | **Fluorescent Channel** |
| --- | --- | --- | --- | --- | --- |
| CD3 | APC-CY7 | 344818 | Biolegend, USA | 638 | 780/60bp |
| CCR7 | AF647 | 557734 | BD pharmingen, USA | 638 | 660/20BP |
| CD8 | FITC | 300906 | Biolegend, USA | 488 | 525/40BP |
| CD19 | PerCPcy5 | 363014 | Biolegend, USA | 488 | 690/50BP |
| CD45RA | BV785 | 304140 | Biolegend, USA | 405 | 780/60BP |
| CD24 | BV650 | 563720 | BD pharmingen, USA | 405 | 660/20BP |
| CD27 | BV605 | 562655 | BD pharmingen, USA | 405 | 610/20BP |
| CD38 | BV421 | 303525 | Biolegend, USA | 405 | 450/45BP |
| L/D | BV510 | 65-0866-14 | Invitrogen, USA | 405 | 525/40BP |
| **Antibody names** | **Fluorescent substance** | **Catalog** | **Manufacturer** | **Laser** | **Fluorescent Channel** |
| CD3 | APC-CY7 | 344818 | Biolegend, USA | 638 | 780/60bp |
| CD8 | PE-CY7 | 557746 | BD pharmingen, USA | 488 | 780/60BP |
| CCR6 | AF700 | 353434 | Biolegend, USA | 638 | 712/25BP |
| CXCR3 | PE | 557185 | BD pharmingen, USA | 488 | 585/42BP |
| PD-1 | BB700 | 566460 | BD pharmingen, USA | 488 | 690/50BP |
| CXCR5 | FITC | 563105 | BD pharmingen, USA | 488 | 525/40BP |
| CD19 | APC | 392504 | Biolegend, USA | 638 | 660/20BP |
| CD38 | BV421 | 303525 | Biolegend, USA | 405 | 450/45BP |
| L/D | BV510 | 65-0866-14 | Invitrogen, USA | 405 | 525/40BP |
| **Antibody names** | **Fluorescent substance** | **Catalog** | **Manufacturer** | **Laser** | **Fluorescent Channel** |
| CD3 | APC-CY7 | 344818 | Biolegend, USA | 638 | 780/60bp |
| CD8 | AF700 | 557945 | BD pharmingen, USA | 638 | 712/25BP |
| CXCR3 | PE | 557185 | BD pharmingen, USA | 488 | 585/42BP |
| IFN-γ | APC | 502512 | Biolegend, USA | 638 | 660/20BP |
| TNF-α | PE-CY7 | 557647 | BD pharmingen, USA | 488 | 780/60BP |
| 1L-21 | BV421 | 564755 | BD pharmingen, USA | 405 | 450/45BP |
| CXCR5 | FITC | 563105 | BD pharmingen, USA | 488 | 525/40BP |
| L/D | BV510 | 65-0866-14 | Invitrogen, USA | 405 | 525/40BP |
|  |  |  |  |  |  |
| **Antibody names** | **Fluorescent substance** | **Catalog** | **Manufacturer** | **Laser** | **Fluorescent Channel** |
| CD3 | APC | 560176 | BD pharmingen, USA | 638 | 660/20BP |
| CD19 | PE-CY7 | 560911 | BD pharmingen, USA | 488 | 780/60BP |
| CD8 | PerCP | 344708 | Biolegend, USA | 488 | 690/50BP |
| CXCR3 | PE | 557185 | BD pharmingen, USA | 488 | 585/42BP |
| CCR6 | AF700 | 353434 | Biolegend, USA | 638 | 712/25BP |
| IFN-γ | BV650 | 502537 | Biolegend, USA | 405 | 660/20BP |
| 1L-10 | BV421 | 564053 | BD pharmingen, USA | 405 | 450/45BP |
| L/D | BV510 | 65-0866-14 | Invitrogen, USA | 405 | 525/40BP |
| **Antibody names** | **Fluorescent substance** | **Catalog** | **Manufacturer** | **Laser** | **Fluorescent Channel** |
| CD3 | APC-CY7 | 344818 | Biolegend, USA | 638 | 780/60bp |
| CD8 | Percp | 344708 | Biolegend, USA | 488 | 690/50BP |
| CD127 | APC | 351316 | BD pharmingen, USA | 638 | 660/20BP |
| CCR6 | AF700 | 353434 | Biolegend, USA | 638 | 712/25BP |
| CD25 | PE | 356104 | Biolegend, USA | 488 | 585/42BP |
| IFN-γ | FITC | 502505 | Biolegend, USA | 488 | 525/40BP |
| TNF-α | PE-CY7 | 557647 | BD pharmingen, USA | 488 | 780/60BP |
| IL-21 | BV421 | 564755 | BD pharmingen, USA | 405 | 450/45BP |
| L/D | BV510 | 65-0866-14 | Invitrogen, USA | 405 | 525/40BP |
| **Antibody names** | | **Catalog number** | | **Manufacturer** | |
| Anti-CDKN2A/p16INK4a antibody - N-terminal | | ab189034 | | Abcam | |
| P21 Polyclonal antibody | | 10355-1-AP | | Proteintech | |
| P53 Polyclonal antibody | | 10442-1-AP | | Proteintech | |
| Beta Actin Polyclonal antibody | | 20536-1-AP | | Proteintech | |

**Supplementary Table 2**. Primer sequences for quantitative real-time PCR (from 5’ to 3’).

| Gene | Direction | Sequence |
| --- | --- | --- |
| p16  (human) | Forward | CCCAACGCCCCGAATAGTTTA |
|  | Reverse | ACCAGCGTGTCCAGGAAG |
| p21  (human) | Forward | AGGGCATCTCCTCCTATCC |
|  | Reverse | CCTCTACTGCCACCATCTT |
| p53  (human) | Forward | GACTGACATTCTCCACTTCTTG |
|  | Reverse | CTTCTTGCGGAGATTCTCATT |

**Supplementary Figure 2** Genes from SenMayo Geneset expression levels on multiple CD4+T cell sub-types spilt by HIV+ treated-naïve and healthy individuals


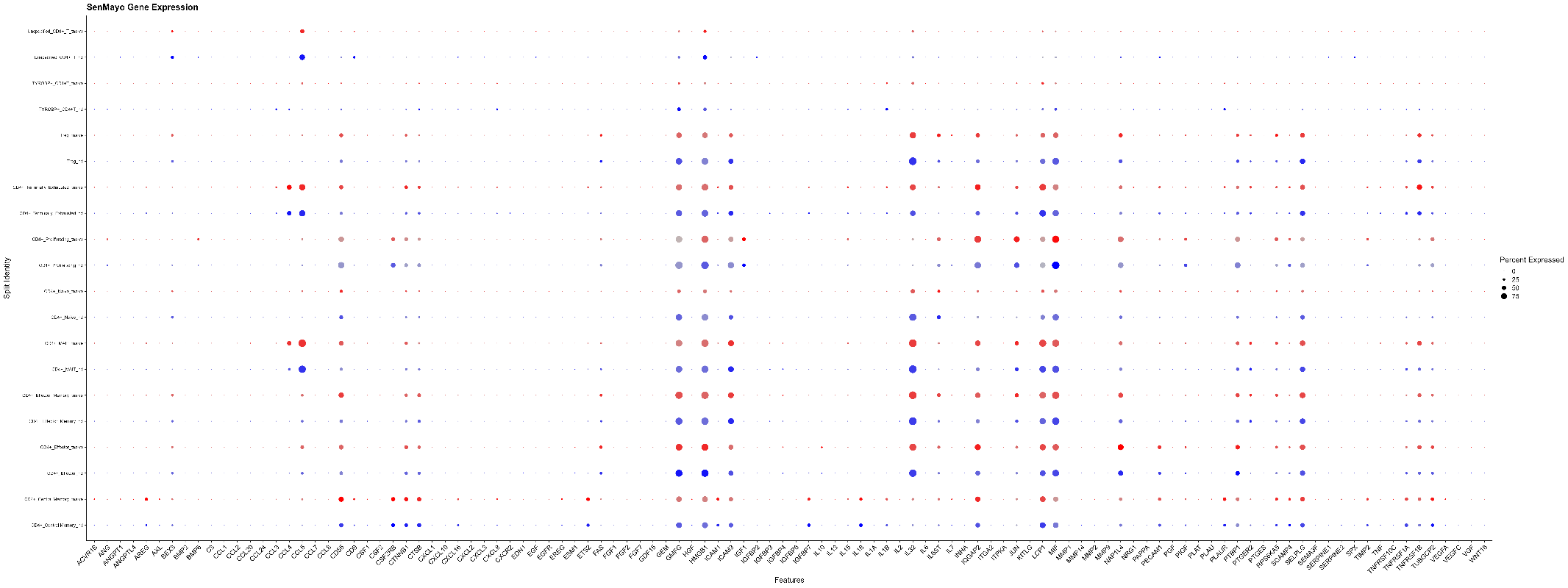


**Abbreviations: tnaive: treated-naïve PLWHIV; hd: Healthy donors.**

**Supplementary Table 3 Baseline Characteristics of PLWHIV for mechanical experiment**

| **Variables** | **PLWHIV in AIDS Stages** | | **P value** | **PLWHIV in non-AIDS Stages** | | **P value** |
| --- | --- | --- | --- | --- | --- | --- |
|  | **NIR** | **IR** |  | **NIR** | **IR** |  |
| **Sample size** | 11 | 12 | - | 12 | 11 | - |
| **Gender, male** | 7 | 7 | 0.832 | 7 | 9 | 0.642 |
| **Age, years** | 44.78±10.46 | 40.27±13.31 | 0.032 | 39.97±9.81 | 36.57±11.42 | 0.035 |
| **Baseline CD4 (Cells/μL)** | 114.63±71.54 | 116.39±60.18 | 0.537 | 427.36±62.87 | 351.92±77.28 | 0.381 |
| **Baseline CD8 (Cells/μL)** | 1172.65±2368.44 | 985.02±621.20 | 0.847 | 1094.53±487.53 | 1214.23±585.77 | 0.124 |
| **Baseline CD4/CD8 Ratio** | 0.10±0.17 | 0.11±0.20 | 0.259 | 0.39±0.27 | 0.30±0.80 | 0.546 |
| **Baseline HIV RNA Viral Load (Log_10_IU/mL)** | 2.31±0.58 | 3.52±0.83 | 0.188 | 3.11±0.72 | 3.57±0.60 | 0.137 |
